# Six Years of Research on the National Institute of Mental Health’s Research Domain Criteria (RDoC) Initiative: A Systematic Review

**DOI:** 10.3389/fncel.2017.00046

**Published:** 2017-03-03

**Authors:** Dean Carcone, Anthony C. Ruocco

**Affiliations:** Departments of Psychology and Psychological Clinical Science, University of TorontoToronto, ON, Canada

**Keywords:** research domain criteria (RDoC), mental disorders, cells, circuits and systems, physiology, brain, neuroscience

## Abstract

Six years have passed since the National Institute of Mental Health (NIMH) in the United States launched the Research Domain Criteria (RDoC) initiative. The RDoC introduces a framework for research on the biology of mental illness that integrates research findings across multiple levels of information. The framework outlines constructs that represent specific quantifiable dimensions of behavior (e.g., responses to acute threat, cognitive control) and corresponding units of analysis that can be used to study the constructs, beginning at the levels of genes, molecules, cells, circuits and physiology, and moving up to behaviors and self-reports. In this systematic review, a literature search was conducted to synthesize empirical research published since the proposal of the framework that incorporated the RDoC. Forty-eight peer-reviewed scholarly articles met eligibility criteria for the review. Studies differed according to whether they analyzed RDoC constructs and units of analysis within vs. between clinically-diagnosed and non-psychiatric samples. The most commonly studied constructs were subsumed within the domains of Negative Valence Systems, Positive Valence Systems and Cognitive Systems, providing initial results which primarily connected genetics, brain circuits and physiology research findings with behavior and self-reports. Prospects for future research adopting the RDoC matrix and utilizing a dimensional approach to studying the biology of mental illness are discussed.

## Introduction

The Research Domain Criteria (RDoC) framework was unveiled by the National Institute of Mental Health (NIMH) in a commentary published in the American Journal of Psychiatry (Insel et al., 2010). In response to concerns over the validity of the diagnostic criteria espoused in the Diagnostic and Statistical Manual (DSM) and what some consider inadequate efforts to address these concerns in the process of revising the manual in the DSM-5, the RDoC was proposed as an alternate framework to conceptually organize and direct biological research on mental disorders (Cuthbert and Insel, 2013; Insel, 2013). This framework encourages research to be structured around five major domains: Negative Valence Systems, driving reactions to aversive stimuli; Positive Valence Systems, driving reactions to positive stimuli; Cognitive Systems, including various mental processes; Social Processes, responsible for interpersonal behavior and cognition; and Arousal and Regulatory Systems, involved in context-based and homeostatic regulation or neural systems. Research on these systems and processes is organized around a dimensional approach incorporating and integrating the following levels or units of analysis: genes, molecules, cells, circuits, physiology, behavior and self-report. By re-orienting research away from DSM categories and toward a multimodal dimensional framework based on empirically validated constructs, the aim of the NIMH’s RDoC initiative has been to progress further understanding of these domains such that a new diagnostic nosology can be developed.

The initial structure and five domains of the RDoC Matrix were the product of a series of workshops arranged by the NIMH (see Development of the RDoC Framework (2016) on the NIMH website). Each of the domains is subdivided into constructs representing specific functional dimensions of behavior. For example, the Cognitive Systems domain encompasses the constructs Attention, Perception, Declarative Memory, Language, Cognitive Control and Working Memory. Some of these constructs are further divided into subconstructs, such as the division of Perception into Visual Perception, Auditory Perception, and Olfactory/Somatosensory/Multimodal Perception. These constructs and subconstructs are crossed with the seven RDoC units of analysis to form the skeleton of the RDoC Matrix. Cells of this matrix are filled with relevant empirically supported *elements*, describing relevant research topics within a construct which can be investigated at a given unit of analysis. For example, dopamine and serotonin are molecular elements of the Reward Valuation subconstruct of Approach Motivation, under the Positive Valence Systems domain. For an illustration of all divisions within the matrix, please refer to The RDoC Matrix (2016) on the NIHM website. The organization of the RDoC Matrix remains flexible, having undergone additions, modifications, and a recent redesign since its launch (RDoC Launches User-Friendly Matrix Format, 2016).

Six years have passed since the proposal of the RDoC framework, conceivably allowing sufficient time for researchers to design, conduct, and publish initial studies that adopt this innovative new framework. The purpose of this brief systematic review is to summarize all peer-reviewed studies published since the proposal of the framework that explicitly purport to incorporate at least one domain, construct, or element of the RDoC. The aim of the review article is to provide an overview of research on the RDoC to identify patterns in the domains, constructs, and units of analysis most commonly studied and the research designs that have been employed.

## Method

### Database Search

**Figure 1:**
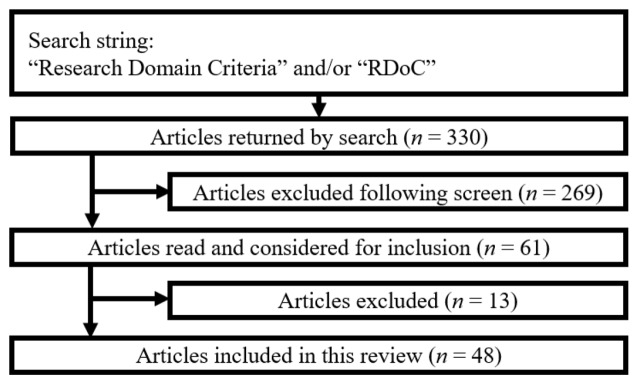
**Flowchart of articles screened and selected**.

The ProQuest search engine was used to access the PsycINFO and Medline databases to return all studies indexed using the term “Research Domain Criteria” and/or the acronym “RDoC”. The results were restricted to peer-reviewed work published in a scholarly journal after April 2013, the date that the RDoC was officially proposed. This search was conducted on April 4, 2016, and returned 330 unique results. Abstracts for these 330 studies were initially screened to remove reviews, commentaries, and results erroneously returned by the search (e.g., those pertaining to Refractory Dissolved Organic Carbon). Following this screen, 61 potentially relevant articles were selected to pass through a second screen following review of the full text of each manuscript. Please refer to Figure 1 for a visual representation of article screening and selection.

### Article Inclusion and Exclusion

In order for an article to be selected for inclusion in this review, it must have met the following inclusion criteria: (1) empirically examined one or more identified RDoC domains, constructs, or elements; and (2) made direct reference to the RDoC framework as part of the study rationale, interpretation of results, or implications and future directions. In order to be retained, an article must also not have met any of the following exclusion criteria: (1) the article represented a proposal for RDoC-related research that had not yet been conducted; (2) the focus of an article was the empirical validation of a psychometric measure related to an RDoC construct, rather than the examination of the construct itself; and (3) the article centered around a case study. Of the 61 articles selected for full-text review, the majority (48 articles) were found to be relevant primary empirical studies which explored themes consistent with the RDoC framework. These articles have been included in this review. A summary of the constructs and units of analysis explored, as well as the key results of these studies, have been included in a Supplementary Table provided with this review article. It should be noted that additional research closely related to RDoC constructs, although not explicitly identifying use of the RDoC framework, has been conducted during the previous 6 years. These studies have not been included in this review article.

## Results

### Common Experimental Designs

Many of the studies reviewed shared similar experimental designs and approaches to applying the RDoC framework. The most common approach was to explore an explicitly defined RDoC construct, subconstruct, or element using two or more units of analysis. This often resulted in describing relationships between higher level behaviors or traits and lower level biological underpinnings. Additionally, many of these studies overtly employed a transdiagnostic approach by examining a given construct across more than one group defined by clinical diagnosis. Another common approach was to explore associations between constructs or subconstructs within or across RDoC domains using one or more units of analysis. Alternatively, a number of studies employed a dimensional approach to study psychological or behavioral phenotypes related to established RDoC domains but focusing on constructs not explicitly defined within the RDoC matrix. Finally, two studies explored the application of the RDoC to the design of clinical interventions. Please refer to the Supplementary Table for additional information on the experimental designs and results of all studies reviewed.

### Arousal and Regulatory Systems

Since the proposal of the RDoC framework, domains have not been equally represented in the literature. With the exception of six studies not specifying a particular domain of focus, almost all the research summarized in this review examined at least one construct in either the Cognitive Systems, Positive Valence Systems, or Negative Valence Systems domains. Only two studies (Tegeler et al., 2015; Olbrich et al., 2016) focused exclusively on the domain of Arousal and Regularity Systems, both targeting the Arousal construct. These studies also both used similar physiological measures to study clinical samples. Tegeler et al. (2015) demonstrated that hemispheric dominance in high-frequency brain activity was related to baroreflex sensitivity and higher resting heart rate. Olbrich et al. (2016) showed that pre-treatment physiological measures predict remission from major depressive disorder (MDD) and response to SSRI treatment. Both of these studies highlight the potential of the RDoC for studying arousal systems relevant to psychopathology.

### Social Processes

Similar to the Arousal and Regulatory Systems, only two studies exclusively examined the Social Processes Domain (Fang et al., 2014; Lindberg et al., 2015). Both of these studies explored the Affiliation and Attachment construct, although the experimental paradigms employed by each were quite distinct. Fang et al. (2014) recorded behavior on a social exclusion task in males with social anxiety disorder following administration of oxytocin. For males with lower self-reported attachment avoidance, oxytocin promoted social affiliation and cooperation. In contrast, Lindberg et al. (2015) described how similar scales of the Attachment and Clinical Issues Questionnaire (Lindberg and Thomas, 2011) are associated with both current alcohol dependence in adults and risk of future alcohol dependence in high school students. Although research has now examined the Affiliation and Attachment construct within the context of the RDoC, the remaining constructs of Social Communication, Perception and Understanding of Self, and Perception and Understanding of Others have yet to be independently explored.

### Positive Valence Systems

Twelve publications included in this review article, examined Positive Valence Systems, although none of these explored the constructs of Habit or Initial/Sustained Responsiveness to Reward Attainment. Rather, research has primarily focused on reward-related constructs such as approach motivation (Karalunas et al., 2014) and reward learning (Webb et al., 2016). For example, Sharp et al. (2014) used fMRI to compare brain activation patterns during a card-guessing reward task between three female groups: depressed adolescents with a maternal history of depression, never-depressed adolescents with a maternal history of depression, and never-depressed adolescents with no maternal history of depression. Lower right ventral striatal activity was observed for both currently depressed and at-risk adolescents. Additionally, activity in this region was inversely correlated with maternal Beck Depression Inventory (BDI) scores across all groups. These results highlight the potential for disruptions in Positive Valence Systems to be used as identifiable vulnerability factors for the development of MDD.

Arrondo et al. (2015) employed a similar transdiagnostic approach in the examination of circuitry related to the Expectancy construct. Patients with schizophrenia, MDD, and controls completed a task to induce anticipation of monetary reward while undergoing fMRI. Reduced activation associated with reward anticipation was observed in the bilateral ventral striatum for both patient groups compared to controls. This reduction in activation was related to self-reported anhedonia and overall depression symptoms for patients with schizophrenia but not MDD. Through their application of the RDoC, these two studies showcase the potential for transdiagnostic and dimensional examination of psychological constructs.

### Cognitive Systems

Research on Cognitive Systems has been considerable and diverse, with eighteen studies published on four of the six constructs within the domain (e.g., Cognitive Control: Lopez-Garcia et al. (2016); Perception: Silverstein et al. (2015); Attention: Kleinman et al. (2015); and Working Memory: Francazio and Flessner (2015)). To elaborate on one example, Chan et al. (2015) demonstrated how a standard performance-monitoring event-related potential paradigm can be used within an RDoC framework to examine Cognitive Control and Performance Monitoring systems. Adults with a history of psychosis and a control group completed a flanker task while undergoing EEG. Reduced error-related negativity and error positivity amplitudes were observed for individuals with a history of psychosis compared to controls. Additionally, reduced error positivity amplitude was associated with schizotypal personality traits across both groups, suggesting that these disruptions in neural circuitry may represent a transdiagnostic phenotype.

As an additional example of a study employing multiple units of analysis to explore a Cognitive System, Newman et al. (2016) focused on the Response Selection construct by examining the relationship between cortical thickness and performance on a go/no-go task. In a sample of 114 adults, 46% of which exhibited symptoms of Attention Deficit Hyperactivity Disorder (ADHD) persisting into adulthood, thickness of the caudal inferior frontal gyrus was associated with poorer response inhibition (i.e., more commission errors) regardless of ADHD symptoms or history of substance abuse. Of note, cortical thickness of this area was also inversely correlated with frequency of cannabis use and persistence of ADHD symptoms.

A transdiagnostic approach to the study of the Visual Perception Cognitive System was adopted by Silverstein et al. (2015). This work investigated disturbances in perceptual organization across patients with body dysmorphic disorder (BDD) and schizophrenia, which are two patient groups defined in part by perceptual distortions. Performance on tasks of perceptual organization was compared between these groups, non-patients, and an obsessive-compulsive disorder (OCD) group. Only patients with schizophrenia performed worse on these measures than comparison groups, while patients with BDD did not differ from non-patients or OCD patients. Consequently, it was suggested that the disturbances in visual perception found in BDD may be unrelated to problems in perceptual organization. Furthermore, these results are consistent with the conclusion that disturbances in perceptual organization may be more specific to schizophrenia and other neurodevelopmental disorders.

Rather than focusing on an RDoC *construct*, one study examined a single *element* of the Perception construct within the Cognitive Systems matrix. Chen et al. (2016) explored the relationship between the expression of the dysbindin protein, found in neural tissue, and disturbances in the regulation of lipid synthesis and synaptic plasticity. A reduction of sterol regulatory element binding protein-1 (SREBP1), a transcriptional regulator for lipid synthesis, was found in both deceased patients with schizophrenia and dysbindin-1 knockout mice. Additional results suggest that this disturbance in SREBP1 maturation may lead to a disruption in synaptic plasticity, and these disruptions may be corrected in knockout mice with the administration of the anti-psychotic drug clozapine. This multi-modal approach illustrates the goals of the RDoC framework, using multiple levels of analysis to provide a comprehensive empirical characterization of clinically relevant phenotypic variation.

### Negative Valence Systems

The domain most commonly explored by the research collected in this systematic review was Negative Valence Systems, with 20 publications examining at least one construct within this domain. With the exception of Frustrative Non-Reward, four of the five constructs within this domain were investigated by at least one study (e.g., Acute Threat: Yancey et al. (2016); Potential Threat: Latzman et al. (2016); Sustained Threat: Weinberg et al. (2016); and Loss: Woody et al. (2014)), with Acute Threat the most commonly studied construct. As an example, Bauer et al. (2013) explored Acute Threat using both physiological and self-report measures. The Clinician Administered Posttraumatic Stress Disorder (PTSD) Scale (Blake et al., 1995) was administered to 36 adults reporting prior exposure to a traumatic event, a sample in which the Acute Threat system may be compromised. Additionally, these adults underwent physiological recordings, including heart rate, skin conductance, and eye-blinks, while performing a script-driven emotional imagery task. Measures of physiological reactivity, thought to be sensitive to disturbances in the Acute Threat system, correlated significantly with severity of PTSD symptoms at an initial visit and at a six-month follow-up assessment. This was taken to suggest that physiological reactivity is a stable and valid measure of disturbances in the Acute Threat construct associated with PTSD. Furthermore, Pineles et al. (2013) demonstrated that these physiological measures are a better predictor of PTSD diagnosis than self-report measures of emotional response.

An additional noteworthy study used a population of 76 captive chimpanzees to investigate the Potential Threat construct as it relates to genes, physiology and anxious behavior (Latzman et al., 2016). It was shown that scratching behavior, an indicator of negative arousal, exhibited a sex-specific association with both AVPR1A genotype, a gene related to mammalian social behavior, and brain morphometry in regions associated with this gene. Although only three studies (Chen et al., 2016; Kondo et al., 2016; Latzman et al., 2016) employed non-human mammals in their research, they highlight how animal research can contribute to the understanding of species-general elements within the RDoC matrix.

A number of studies explored topics related to established domains, but focused on constructs not explicitly defined as a construct in the RDoC matrix, such as neuroticism (i.e., the tendency to experience negative affect; Bedwell et al., 2014) and anhedonia (i.e., the inability to feel pleasure; Webb et al., 2016). One of these studies, Østergaard et al. (2014), overtly proposed that melancholia should be included as an additional construct within the Negative Valence Systems domain. Analysis of self-report measures of depression symptoms in a large treatment study of depression suggested a common and unidimensional construct reflecting melancholia is sensitive to pharmacological intervention. According to the authors, the results of this and prior work highlight how melancholia meets the criteria for inclusion as an RDoC construct, as stipulated in Cuthbert and Insel (2013). As the RDoC matrix is a work in progress, it will be interesting to see how proposals like this can influence its development.

### Research Exploring More than One Domain

Ten of the studies reviewed examined more than one RDoC domain, commonly exploring associations between constructs or subconstructs across multiple domains. Verona and Bresin (2015), for example, examined the relationship between threat-related Negative Valence Systems and the Cognitive System subconstruct of Response Selection. Adults with a history of violent and criminal offences completed the Buss Perry Aggression Questionnaire (Buss and Perry, 1992), which was taken as an index of proneness to aggression associated with Sustained and Acute Threat responses. Participants then underwent EEG while completing an emotional-linguistic go/no-go task. During blocks presenting threatening words, self-reported anger and aggressive behavior each were associated with smaller P3 amplitude recorded during response inhibition, an electrophysiological marker that is taken to represent reduced inhibitory control processing. It is suggested that this relationship may indicate that sensitivity to acute or sustained threat may deteriorate cognitive control processes.

An additional example of research spanning across multiple domains is work which explored the application of the RDoC to the design of clinical interventions. “Engage”, first described in Alexopoulos and Arean (2014), is a psychological intervention designed to specifically target disturbances in RDoC-defined Positive and Negative Valence Systems through *reward exposure*, the facilitation of meaningful and rewarding activities. A sample of older adults with MDD received 9 weeks of Engage (Alexopoulos et al., 2015). Symptom improvements within this group were compared to a patient group from a previous study (Alexopoulos et al., 2011), in which participants received problem-solving therapy (PST). The Engage intervention demonstrated comparable efficacy to PST in reducing symptoms and functional impairment associated with MDD. In order to explore the mechanism underlying this change, Alexopoulos et al. (2016) examined how MDD symptoms and a self-reported rating of behavioral activation influenced each other throughout Engage. In a sample of 48 older adults with MDD completing 9 weeks of Engage, greater increases in behavioral activation, taken as a broad index of Positive Valence Systems function, predicted change in depression severity during treatment and at a 36-week follow-up assessment. These investigations may mark the first of many treatment studies based on targeting dimensions included in the RDoC Matrix.

## Discussion

With 6 years having elapsed since the RDoC framework was introduced, it is timely to review findings from the first wave of research which adopted the framework. This systematic review identified 48 primary empirical articles published in the last 4 years (see Supplementary Table) which either directly examined an RDoC-defined construct, or interpreted their results in a manner consistent with the RDoC framework. As described above, the majority of these studies fall into common prototypes regarding their goals and their approach to applying the RDoC framework. At present, it seems that an “RDoC study” describes: (1) research on a single defined RDoC construct using multiple levels of analysis; (2) research which explores associations between separate RDoC constructs and/or elements; and/or (3) research which adopts a transdiagnostic perspective to investigate an RDoC construct by examining multiple categories of a disorder or symptom dimensions. Some additional studies purport to use approaches consistent with the RDoC framework but incorporate phenotypes which are not (or may not yet have been) specified within the RDoC matrix. We do not know if RDoC studies will continue to fall within these rough categories, but the approaches that they represent are informative for the design of subsequent research. The topics of focus for these future studies will likely be influenced by the growth and modification of the RDoC matrix and by significant advances in research made within this framework. Whether this pattern of research reflects the way future studies will be designed likely depends on subsequent modifications to the RDoC matrix and the progression of research during these formative years of the framework.

This systematic review highlights a notable scarcity of research explicitly intended to examine the RDoC domains of Social Processes and Arousal and Regulatory Systems. This is of course not to say that research in these and related areas has not been carried out but that the RDoC framework specifically has not been adopted in publications of this work. Conversely, a remarkable number of studies have been published which explore the domains of Cognitive Systems, Negative Valence Systems, and Positive Valence Systems. It is unlikely that this disparity is an artifact of the database search used in this review, as no domain-specific terms were included as search criteria. It may be speculated that constructs related to the more well-represented domains may currently be easier to cast within the RDoC framework, prompting a greater number of researchers to incorporate this new perspective. For example, research on attention may lend itself more easily to be examined from a multi-level biological and behavioral perspective, while work of this type may be less common when examining topics such as self-perception or non-verbal communication. Different fields of study may also have been quicker or slower to adopt the RDoC framework depending at least in part on the established theories in the field that emphasize or deemphasize biological contributions to the constructs of interest. This may be reflected in the relative number of unoccupied cells in the lower-level units of analysis present within the RDoC matrix at the time of this review (e.g., Cognitive Systems: 17% unoccupied vs. Social Processes: 34% unoccupied; see The RDoC Matrix (2016)). However, this does not explain the lack of research using the RDoC framework to investigate Arousal and Regulatory Systems, a domain that is well populated with respect to RDoC units of analysis. Rather, it can be argued that this domain is historically better understood than other domains (although perhaps not in relation to self-report constructs relevant to mental and neurological illness), and consequently has been the focus of fewer research studies since the introduction of the RDoC Framework only 6 years ago.

Although the transition to a dimensional and empirically-driven research framework on mental illness has been anticipated for many years (Krueger and Piasecki, 2002), the RDoC proposal has been met with a mixture of support, resistance and controversy. For a discussion of the challenges facing the RDoC framework, please refer to Patrick and Hajcak (2016). It has been suggested that all authoritative systems of nosology (including both the DSM and the RDoC) likely impeded the development of scientific theory by constraining competitive discourse (Markon, 2013). Additionally, having more than one active system of nosology may promote fractures in research continuity between funding bodies and geographic regions. Although researchers in the United States are in part incentivized to adopt an RDoC framework by receiving funding from the NIMH, researchers outside the region may not yet have as strong of an incentive to do so and therefore may be less likely to make the transition. Conversely, many researchers are optimistic about the promise of dimensional approaches to studying mental illness, components of which are inherent to many aspects of the RDoC framework. Indeed, there is potential for the framework to provide a useful alternative to structuring clinical research on mental illness (Casey et al., 2013), although the existing body of research incorporating this framework is relatively small and requires further advancement to refine the model and realize the potential of this transformative new approach.

## Author Contributions

DC and ACR conceptualized the topic, reviewed studies for eligibility in the review, and wrote the manuscript. DC conducted the literature search.

## Funding

ACR is supported by a New Investigator Salary Award (MSH-130177) from the Canadian Institutes of Health Research and an Early Researcher Award (ER14-10-185) from the Ministry of Research and Innovation, Province of Ontario. DC is supported by an Ontario Graduate Scholarship.

## Conflict of Interest Statement

The authors declare that the research was conducted in the absence of any commercial or financial relationships that could be construed as a potential conflict of interest.
