# Supplementary material for: Six Years of Research on the National Institute of Mental Health’s Research Domain Criteria (RDoC) Initiative: A Systematic Review
Source: Front Cell Neurosci. 2017 Mar 3;11:46. doi: 10.3389/fncel.2017.00046 (PMC5334510; doi:10.3389/fncel.2017.00046)
Supplement: Supplementary file 1 [file Table_1.docx]

**Supplementary Table**

| Citation | Domain | Construct(s)/ Subconstruct(s) | Unit(s) of analysis. | Focus/ Paradigm(s)/ Element(s) | Sample | Key Findings |
| --- | --- | --- | --- | --- | --- | --- |
| Olbrich et al., 2016 | Arousal and Regulatory Systems | Arousal | Physiology | EEG and ECG during rest prior to antidepressant treatment | 23 patients with MDD, 598 MDD patients from the iSPOT-study | MDD remission and response following SSRI treatment was associated with a faster decline of arousal at baseline. For SNRI treatment, a larger increase in arousal at baseline predicted positive response. |
| Tegeler et al., 2015 | Arousal and Regulatory Systems | Arousal | Physiology; | Heart rate variability, baroreflex sensitivity, EEG to detect hemispheric asymmetry (HIRREM assessment) | 131 adults with mixed clinical diagnoses | Individuals exhibiting right hemispheric dominance in high-frequency brain activity demonstrated lower baroreflex sensitivity and higher resting heart rate. |
| Kondo et al., 2016 | Arousal and Regulatory Systems; Cognitive Systems; Negative Valence Systems; Social Processes | Declarative Memory; Perception; Cognitive Control; Acute Threat; Potential Harm; Sustained Threat; Affiliation and Attachment; Arousal | Behaviour | Multiple indices, including exposure responses, latency to feed, locomotor activity, etc. | Mice having undergone short-term exposure to cuprizone or a control substance | The results suggest that the cuprizone short-term exposure mouse may present a useful model of the mechanisms associating inflammation and psychosis, allowing the study of a number of RDoC domains and constructs characterized in these mice. |
| Wu et al., 2016 | Arousal and Regulatory Systems; Cognitive Systems; Positive Valence Systems; Social Systems | Arousal; Cognitive Control; Attention; Declarative Memory; Working Memory; Social Communication; Reward-Related Processed | Physiology; behaviour | Diffusion weighted imaging; neurocognitive tasks (affective go/no-go, Cambridge gambling task) | 70 patients with a bipolar disorder, 38 healthy controls | Unsupervised machine learning suggested two phenotypically distinct groups within the bipolar group according to neurocognitive performance. The group demonstrating poorer performance also exhibited distinct FA and MD from the other phenotypic group and the control group, which did not significantly differ on these measures/ |
| Chan et al., 2015 | Cognitive Systems | Cognitive Control; Performance Monitoring. | Physiology; self-report | ERPs underlying error monitoring; SPQ-BR | 14 adults with a history of psychosis, 12 controls | Reduced error-related negativity and error positivity was observed for adults with a history of psychosis. Reduced error positivity was related to Schizotypal personality traits across both groups. |
| Chen et al., 2016 | Cognitive Systems | Element: Dysbindin | Genes; molecules; cells; circuits | Dysbindin-1 gene; DTNBP1, SREBP1, ARC; PC12 cells, murine hippocampal culture cells, post-mortem human hippocampal cells; in vivo/vitro hippocampal activity | 25 deceased adults, 13 with schizophrenia. Various murine samples and cell cultures | SREBP1 expression is reduced in both dysbindin-1 knockout mice and postmortem brain tissue of human patients with schizophrenia. Additional results suggest that interactions between DTNBP1, SREBP1, and ARC may be related to the cognitive deficits present in schizophrenia. |
| Patrick et al., 2013 | Cognitive Systems | Cognitive Control; Inhibition/ Suppression | Physiology; self-report; | EEG using visual oddball task; ESI; interview | 393 or 566 community-dwelling adults | ESI scores predict a variety of externalizing problems and correlate with neural indices of disinhibition proneness. These can be combined to form an effective psychoneurometric predictor. |
| Francazio & Flessner, 2015 | Cognitive Systems | Working Memory; Flexible Updating | Behaviour; self-self-report | IDED Test; DASS-21, PI-WSUR, MGH-HS, SPS | 56 undergraduate students, some with obsessive-compulsive behaviours | Cognitive flexibility was lower in students with obsessive-compulsive behaviours compared to controls. Cognitive flexibility did not predict severity of obsessive-compulsive behaviour. |
| Kleinman et al., 2015 | Cognitive Systems | Attention | Behaviour; self-report | CPT-II; CGAS, CDRS-R, YMRS, MTA SNAP-IV | 23 child and adolescents with ADHD, 10 patients with a bipolar disorder, 33 patients with ADHD and bipolar disorder, and 18 controls | Cluster analysis divided individuals into two groups based on performance on the CPT-II. ADHD and ADHD+bipolar patients were divided between groups, while control and bipolar-only patients were primarily assigned to one (high performance) group. The group with poor performance also exhibited higher functional impairment. |
| Lopez-Garcia et al., 2016 | Cognitive Systems | Cognitive control | Circuits, behaviour | fMRI during AX-CPT and DPX-CPT, functional connectivity; behavioural performance | 26 adults without psychiatric diagnoses | Activation in similar brain regions was observed for letter and dot pattern continuous performance tasks. Differences included greater engagement of the frontal opercular insula for letters and greater PFC-MTL connectivity for dots patterns. |
| Moser et al., 2015 | Cognitive Systems; Negative Valence Systems; | Acute Threat; Performance Monitoring | Physiology; behaviour; self-report | EEG measures: error-related negativity, resting asymmetry, fear-potentiated startle; Child Behavior Checklist, Child Behavior Questionnaire | 31 children (ages 3 to 7) | Higher fear behaviours were associated with fear-potentiated startle magnitude and right parietal brain activity. Lab-assessed fear-proneness was also associated with smaller error-related negativity responses. |
| Nelson et al., 2016 | Cognitive Systems; Negative Valence Systems; | Acute Threat; Cognitive control: response selection: inhibition/suppression. | Self-report | TF-55; ESI, IDAS, SCID | 471 adults in the community | Both threat sensitivity and weak inhibitory control were associated with internalizing disorder symptoms. Threat sensitivity was more predictive of fear disorders and weak inhibitory control was more predictive of distress disorders and exclusively predictive of substance-related problems. |
| Newman et al., 2015 | Cognitive Systems | Attention; Response Selection | Physiology; Behaviour; self-report | Structural MRI; Go/No-Go; CAARS, SUQ | 114 adults with and without ADHD | The cortical thickness of caudal inferior frontal gyrus was associated with poorer go/no-go performance, not mediated by ADHD. Persistence of ADHD symptoms and frequency of cannabis use were associated with decreased cortical thickness. |
| Ryan et al., 2015 | Cognitive Systems | Cognitive Control | Circuits; behaviour; self-report | Event-related functional MRI during parametric go/no-go embedded within a list learning task; parametric go/no-go; DIGS | 266 female adults with MDD, 202 adults with a bipolar disorder, and 150 healthy controls. 52 female adults underwent MRI | Patients with mood disorders exhibited poorer performance on the cognitive control task than controls. Relatively few areas of shared activation between clinical and control groups were revealed while performing the task. |
| Silverstein et al., 2015 | Cognitive Systems | Visual Perception | Behaviour; self-report | Contour integration test, Ebbinghaus illusion test; BABS, PDI, Y-BOCS, BDD-YBOCS, PANSS | 20 outpatients with BDD, 20 outpatients with OCD, 24 inpatients with schizophrenia, and 20 controls | Only patients with schizophrenia performed significantly worse than controls on tasks of perceptual organization, suggesting that visual processing impairments in BDD may arise from cognitive disturbances in areas other than perceptual organization. |
| Verona & Bresin, 2015 | Cognitive Systems; Negative Valence Systems; | Acute Threat, Sustained Threat; Cognitive control, inhibition/suppression. | Physiology; self-report | EEG recordings during an emotional-linguistic go/no-go task; Buss-Perry Aggression Questionnaire | 67 adults with history of violence and criminal offences | Reduced inhibitory control processing (smaller no-go P3) during presentation of relevant-threat word blocks was associated with tendency toward angry and aggressive behaviour. |
| Karalunas et al., 2014 | Cognitive Systems; Negative Valence Systems; Positive Valence Systems | unspecified; approach motivation; attention | Circuits; physiological; behaviour; self-report | Temperament ratings, physiological recordings, resting state fMRI | 437 children with and without ADHD | Typology based on cardiac physiological response, resting state brain connectivity, and clinical outcome accounted for a greater degree of heterogeneity in the ADHD population than nosologic criteria. |
| Webb et al., 2016 | Cognitive Systems; Negative Valence Systems; Positive Valence Systems | Unspecified (neuroticism); reward learning; cognitive control. | Physiology; behaviour; self-report | Source localization of EEG resting data; PRT, flanker task; HRSD, NEO-FFI-3 | 82 unmedicated adults with MDD | Neuroticism was associated with increased resting gamma current density in the ventral ACC and OFC. Reduced cognitive control was related to decreased gamma activity in the left dlPDC, decreased theta and alpha2 in the dACC, and increased alpha2 in the right dlPDC. Blunted reward learning was associated with lower OFC and left dlPFC gamma activity. |
| Bedwell et al., 2014 | Cognitive Systems; Positive Valence Systems | Unspecified (anhedonia); Declarative Memory. | Self-report; behaviour | ACIPS, TEPS-ANT, TEPS-CON, RBANS immediate and delayed recall | 56 mixed psychiatric and non-psychiatric community-dwelling adults | Decreased social and anticipatory pleasure was associated with psychiatric diagnoses. Memory performance was negatively associated with anhedonia for adults with a history of psychosis. |
| Costa Dias et al., 2015 | Cognitive Systems; Positive Valence Systems; | Reward processing (broadly); attention | Circuits; behaviour | Resting-state functional connectivity using fMRI; delay discounting task, TMCQ | 106 children with and without ADHD | The results suggest that functional connectivity can be used to classify children with and without ADHD into distinct subgroups based on impulsiveness traits. |
| Bauer et al., 2013 | Negative Valence Systems | Acute Threat | Physiology; self-report | Script-driven imagery; CAPS | 36 adults exposed to a (DSM-IV) traumatic event | Physiological reactivity to script-driven imagery was shown to be a stable and valid measure of PTSD symptoms. |
| Lang et al., 2016 | Negative Valence Systems | Acute Threat | Physiology; self-report | Fear-potentiated startle, heart rate, skin conductance, facial muscle action; ASI, BDI-II, MASQ, STAI, STAXI, FSS | 425 treatment-seeking adults with mixed diagnoses | When patients are divided into quintiles based on defensive reactivity, this dimension is associated with diminishing startle reflex and heart rate. Different diagnoses were present across quintiles, highlighting the importance of this trans-diagnostic dimension. |
| MacNamara et al., 2015 | Negative Valence Systems | Unspecified | Physiology, self-report | Late positive potential recorded using EEG; IMAS | 97 outpatients with MDD or GAD, 25 healthy controls | MDD diagnosis and dimensional depression scores were associated with reduced change in late positive potentials following emotional stimuli. GAD diagnosis and dimensional scores were associated with increased change in late positive potentials when controlling for MDD symptoms. |
| Nelson et al., 2015 | Negative Valence Systems | Acute Threat; Potential Threat | Physiology; self-report | Startle eye blinks and ERP responses to acoustic startle probes; Anxiety Sensitivity Index-3 | 131 undergraduate students | Physical concerns were associated with heightened startle response for unpredictable probes and with increased p300 suppression. Cognitive concerns were associated with attenuated startle responses and N100 enhancement for unpredictable probes. |
| Østergaard et al., 2014 | Negative Valence Systems | Melancholia (proposed) | Self-report | HAM-D, IDS-C, QLRS-Q | 2242 patients from the STAR*D study | HAM-D6 and IDS-C5 melancholia scales reflect a unidimensional coherent construct sensitive to specific pharmacological intervention, suggesting this should be included as an RDOC construct. |
| Pineles et al., 2013 | Negative Valence Systems | Unspecified | Physiology; self-report | Script-driven imagery; self-report emotion scales | 150 adults exposed to a (DSM-IV) traumatic event, 78 with PTSD | Reactivity to trauma-related scripts was a better predictor of PTSD diagnosis than self-report measures of emotional response, although both convey partially unique information. |
| Siegle et al., 2015 | Negative Valence Systems | Loss | Physiology; behaviour; self-report | Pupillary reactivity after negative words; emotion-identification/ digit sorting task; measures of loss (including questions from the RRQ, BDI-II, and PANAS) | 84 adults of mixed clinical status and diagnosis | Feeling-related negative affect constructs were associated with prolonged pupillary reactivity during distraction after a negative stimulus. History of abuse was associated with decreased physiological reactivity. These results lend support to the validity of the RDoC loss construct across the levels of analysis tested. |
| Woody et al., 2014 | Negative Valence Systems | Loss | Genes; physiology; self-report | COMT genotype; Heart rate variability; RRS; BDI-II | 97 women, some with a history of MDD or mixed psychiatric disorders | Genotypic variation was associated with heart-rate variability and brooding rumination among women with a history of MDD. |
| Norrholm et al., 2015 | Negative Valence Systems | Acute Threat | Physiology; self-report | Extinction of fear-potentiated startle; modified PSS, TES | 269 adults, some with history of trauma | Fear-potentiated startle response during early extinction was associated with frequency of intrusive distressing thoughts. |
| Yancey et al., 2015 | Negative Valence Systems | Acute Threat | Physiology; self-report | Aversive startle blink response; Trait fear inventory | 421 adult twins | Threat sensitivity predicted enhanced startle reactivity. This relationship was moderated by depression/distress disorders. Among patients with fear disorders, the absence of MDD or distress disorders was associated with reduced startle reactivity. |
| Latzman et al., 2016 | Negative Valence Systems | Potential Threat | Genes, physiology, behaviour, | AVPR1A; VBM using MRI; scratching behaviour | 76 captive chimpanzees | Brain regions where gray matter volume was related to ACPR1A genotype exhibited sex-specific associations with scratching behaviour. |
| Weinberg et al., 2016 | Negative Valence Systems | Sustained Threat | Physiology; self-report | EEG during error monitoring; IDAS-II | 515 never depressed adolescent females | Self-reported checking behaviour is associated with a larger ERN for older adolescents. Depressive symptoms are associated with a smaller ERN irrespective of age. |
| Yancey et al., 2016 | Negative Valence Systems | Acute Threat | Physiology; self-report | Startle response, EEG, facial EMG, heart rate during emotional, picture presentation; TF-55, SCID | 454 adult twins | Physiological indicators of negative emotional reactivity can be combined with self-reported fear measures to create a comprehensive dimension with predictive and discriminant validity related to other physiological and diagnostic factors. |
| Alexopoulos et al., 2015 | Negative Valence Systems; Positive Valence Systems | Reward processing (broadly), otherwise unspecified. | Self-report | SCID; HAM-D; WHODOS; MSSE | 39 older adults with MDD. | Engage, a therapeutic approach designed to target disturbances on RDoC-defined positive and negative valence systems, demonstrated comparable efficacy to Problem Solving Therapy in reducing symptoms of MDD and disability scores. |
| Alexopoulos et al., 2016 | Positive Valence Systems | Reward processing (broadly) | Self-report | HAM-D and BADS administered at baseline, mid-treatment, end of treatment, and at follow-up | 48 older adults with MDD | Change in BADS scores and time predicted subsequent HAM-D scores, suggesting that during Engage therapy improvement in depressive symptoms follows increased behavioural activation. |
| Arrondo et al., 2015 | Positive Valence Systems | Expectancy | Circuits; self-report | Monetary Incentive Delay during fMRI; BPRS; PANSS; SANS; BDI, SHAPS; TEPS | 22 patients with schizophrenia, 24 patients with MDD, 21 controls | Reduced activation in the left and right ventral striatum was observed for both clinical groups compared to controls. This reduction was related to anhedonia and symptoms of depression in patients with schizophrenia but not patients with MDD. |
| Fang et al., 2014 | Social Processes | Affiliation and Attachment | Molecules; behaviour; self-report | Social exclusion (Cyberball), Posner Task | 60 adult males with social anxiety disorder | Following administration of oxytocin, only men with low attachment-avoidance displayed more social affiliation and cooperation, while those with high attachment avoidance displayed faster detection of disgust and neutral faces. |
| Gruber et al., 2015 | Positive Valence Systems | Unspecified (emotional systems) | Physiology; behaviour; self-report | Heart rate variability, heart rate, respiration rate, skin temperature; gross somatic movement, BRMS; mDES | 21 adults with bipolar I disorder, 17 adults with MDD, and 28 controls | Higher intra-individual variation in heart rate was observed for patients with bipolar I disorder. Across groups, heart rate variability was associated with clinician-rated manic symptoms, but not measures of positive affectivity. |
| Hartmann et al., 2014 | Unspecified | Unspecified | Self-report | BABS; EDE; SCID-I/P, BDD-YBOCS, BDI-II, BDIQ | 24 adults with anorexia nervosa, 23 adults with body dysmorphic disorder, and 22 controls | Common endophenotypes were observed between disorders, including lower self-esteem and higher perfectionism in anorexia nervosa. Depression also acted as a mediator between symptom severity and self-esteem in both clinical groups. |
| Lindberg et al., 2015 | Social Processes | Affiliation and Attachment | Self-report | ACIQ, CUGE screen for alcohol abuse, F-MAST, M-MAST | 27 patients with alcohol dependence, 122 controls; 162 high school students | Patients with alcohol dependence exhibited higher scores on a number of ACIQ scales than controls. For high-school students, measures of predicted alcohol dependence were correlated with similar ACIQ scales. |
| Marquand et al., 2016 | Positive Valence Systems (assumed) | Reward-related processes | Circuits, behaviours, self-report | fMRI during a gambling task; delay-discounting task; the Achenbach Adult Self Report instrument | 491 “healthy” adults. | Using normative modeling, the degree to which a participant's brain activity deviated from normal was related to self-reported hyperactivity. These results support the framework that disease can be considered an extreme of a normal range of symptoms. |
| Sharp et al., 2014 | Positive Valence Systems | Reward processing (broadly) | Genes (maternal history); Circuits; Self-report | MFQ; MASC; BDI; card guessing reward task using fMRI | 52 adolescent females with or without depression, with or without maternal history of MDD | Right ventral striatum activation was reduced for both currently depressed and high-risk adolescents. This activation was correlated with maternal depression scores. These results suggest that the Positive Valence Systems domain should be taken as a vulnerability factor for MDD. |
| Stringaris et al., 2015 | Positive Valence Systems | Reward processes (and anhedonia) | Circuits; self-report | fMRI during anticipation of reward in the monetary incentive delay task; DAWVA, SDQ, ADRS | 1576 adolescents with or without current or subthreshold MDD | Subthreshold and clinical MDD was related to reduced activity in the ventral striatum. Transition from subthreshold to clinical depression (at a 2-year follow-up) was predicted by this reduction in activity. Reduced ventral striatum activity was correlated with anhedonia but not low mood. |
| Bebko et al., 2015 | Unspecified | Unspecified | Circuits; behaviour; self-report | Resting-state functional connectivity using fMRI; PGBI-10M, K-DRS, K-MRS, SCARED | 42 patients with depression, 18 controls | Resting state connectivity between the amygdala and posterior insula was negatively associated with behavioural and emotional dysregulation severity and symptoms of depression. Connectivity was not related to diagnostic categories. |
| Jones et al., 2015 | Unspecified | Unspecified | Physiology; self-report | Salivary cortisol awakening response; QIDS | 25 inpatients with depression | Cortisol awakening response (area under the curve increase) at admission predicted treatment response. |
| Leventhal et al., 2016 | Unspecified | Unspecified | Behaviour; self-report | Cigarette and substance use, assessed by survey; RCADS, MDQ, DTS, CASI, SHAPS, TCI, UPPS-P, EATQ-R; DAST, CAST, RAPI | 3310 adolescents | Compared to those using only conventional cigarettes, adolescents using e-cigarettes exhibited fewer internalizing syndromes and multiple transdiagnostic features, including distress tolerance, anxiety sensitivity, and rash actions during negative affect. Use of any type of cigarette was related to poor inhibitory control and impulsivity. |
| Van Meter & Youngstrom, 2015 | Unspecified | Unspecified | Self-report | TEMPS-A, BIS/BAS scales, BDI, HCL-32 | 758 adults, 23 treated for a bipolar disorder, 21 treated for MDD | Temperament style accounted for 49% of the variance in BDI scores within the sample. BAS Fun and Reward scales, cyclothymic temperament, and hyperthymic temperament accounted for 21% of the variance in HCL-23 scores. |
| Webb et al., 2014 | Unspecified | Unspecified | Self-report; physiology; | BDI; PAI; structural MRI followed by VBM | 54 healthy participants | Higher BDI scores and PAI scores were associated with gray matter reductions in four common brain regions: left mOFC and ACC, left thalamus, right superior medial frontal gyrus, and right superior temporal gyrus. |
|  |  |  |  |  |  |  |

Notes. ACIPS: Anticipatory and Consummatory Interpersonal Pleasure Scale Total Score, ACIQ: Attachment and Clinical Issues Questionnaire,

AUQ: Substance Use Questionnaire, AX-CPT: Expectancy Performance Test, BABS: Brown Assessment of Beliefs Scale,

BDD-YBOCS: Yale-Brown Obsessive Compulsive Scale Modified for Body Dysmorphic Disorder, BDI-II: Beck Depression Inventory, BDIQ: Body Image Disturbance Questionnaire, BIS/BAS: Behavioural Inhibition System/Behavioural Activation System, BPRS: Brief Psychiatric Rating Scale, BRMS: Bech-Rafaelsen Mania Scale. CAARS: Conners’ Continuous Performance Test, CAPS: Clinician-Administered PTSD Scale, CASI: Childhood Anxiety Sensitivity Index, Snaith-Hamilton Pleasure Scale, Temperament and Character Inventory (TCI)-Impulsivity,

CAST: Cannabis Abuse Screening Test, CDRS-R: Children’s Depression Rating Scale-Revised, CPT-II: Conners’ Adult ADHD Rating Scale,

DASS: Depression Anxiety and Stress Scales, DAST: Drug Abuse Screening Test-Adolescent Version, DIGS: Diagnostic Interview for Genetic Studies, DPX: Dot Probe Expectancy, DTS: Distress Tolerance Scale, EATQ-R: The Early Adolescent Temperament Questionnaire e Revised,

EDE: Eating Disorder Examination, ESI: Externalizing Spectrum Inventory, IDAS: Inventory of Depression and Anxiety Symptoms

FA: fractional anisotropy, F-MAST: Father Michigan Alcohol Screening Test, M-MAST: Mother Michigan Alcohol Screening Test,

FSS: Fear Survey Schedule, HAM-D: Hamilton Depression Rating Scale, HCL-32 Hypomanic Checklist, HIREM: high resolution, relational, resonance-based electroencephalic mirroring, IDED: Intradimensional/Extradimensional Shift Test, IDS-C: Inventory of Depressive Symptomatology, IMAS: Interview for Mood and Anxiety Symptoms, K-DRS: Kiddie Schedule for Affective Disorders and Schizophrenia for School-Age Children Present Episode Depression Rating Scale, MD: mean diffusivity, MDQ: Mood Disorder Questionnaire, MGH-HS: Massachusetts General Hospital—Hairpulling Scale, MTA SNAPS-IV: Multimodal Treatment of ADHD Swanson Nolan and Pelham Scale,

PANSS: Positive and Negative Syndrome Scale, PDI: the Peters’ Delusional Inventory (PDI), PHBI-10M: Parent General Behavior Inventory-10 Item Mania Scale, PI-WSUR: Padua Inventory—Washington State University Revision, PTSD Symptom Scale, Traumatic Events Inventory,

QIDS: Quick Inventory of Depressive Symptoms scale, QLRS-Q: Quality of Life Enjoyment and Satisfaction Questionnaire, RAPI: Rutgers Alcohol Problem Index, RCADS: Revised Children's Anxiety and Depression Scale, RRQ: Rumination-Reflection Questionnaire, RRS - Ruminative Response Scale, SANS: Scale for the Assessment of Negative Symptoms Beck Depression Inventory, SCARED: Screen for Child Anxiety Related Emotional Disorders, K-MRS: Kiddie Schedule for Affective Disorders and Schizophrenia for School-Age Children Mania Rating Scale, SCID: Structured Clinical Interview for DSM-IV, SHAPS: Snaith–Hamilton Pleasure Scale, SPQ-BR: Schizotypal Personality Questionnaire-Brief Revised, SPS: Skin Picking Scale, STAI: State-Trait Anxiety Inventory, STAXI: State-Trait Anger Expression Inventory,

TEPS: Temporal Experience of Pleasure Scale, TEPS-ANT: Temporal Experience of Pleasure Scale Anticipatory Subscale, TEPS-CON: Temporal Experience of Pleasure Scale Consummatory Subscale, TF-55: 55-item Trait Fear inventory, TMCQ: Temperament in Middle Childhood Questionnaire, Y-BOCS, Yale-Brown Obsessive Compulsive Scale, YMRS: Young Mania Rating Scale.
